# Assessing the Impact of Aging on Burden of Disease

**Published:** 2018-07

**Authors:** Eun-Jung KIM, Sung-Won JUNG, Young-Eun KIM, Dun-Sol GO, Seok-Jun YOON

**Affiliations:** 1. Dept. of Nursing, Pyeongtaek University, Gyeonggi-do, Republic of Korea; 2. Dept. of Nursing, Fareast University, Chungcheongbuk-do, Republic of Korea; 3. Dept. of Preventive Medicine, College of Medicine, Korea University, Seoul, Republic of Korea

**Keywords:** Aging, Disease burden, Difference in difference

## Abstract

**Background:**

In this study, we presented a theoretical model to measure aging rate in OECD countries, quantitatively measuring the effect of aging rate on disease patterns in each country and explaining how these effects were obtained. The purpose of this study was to investigate how disease burden varies according to the level of medical infrastructure and changes in aging index using OECD aging data and WHO disease burden data.

**Methods:**

This study used OECD Health data and global burden of disease data from the WHO in 2000 and 2012. We applied a difference-in-differences (DID) model was used to analyze effects of aging.

**Results:**

Disease burdens increased over time, especially in the aging population of middle-aged. In the case of loss of life due to premature death, the number of middle-aged and older population was increased significantly. When we examined the econometric model after controlling related factors, there was a significant increase in loss of life due to illness and premature death. On the other hand, the group of piles at the aging level had a significant positive effect on Years of Life Lost (YLL). Although the interaction effect as an important variable showing double difference effect of aging did not affect Disability adjusted Life Year (DALY), it showed a significant positive effect on YLL.

**Conclusion:**

Loss of life due to death of the elderly was relatively higher than that of the elderly. Therefore, the impact of population aging on medical resources and medical expenditures in the future should consider population structure changes, disease burden by age group, and interactions of these two incremental factors.

## Introduction

58.2% of survey participants (n=2,549) who were older than 65 suffer from a chronic disease, supporting the estimation that the prevalence of chronic diseases was high among the elderly (1). Such increase in prevalence of chronic diseases might be accelerated due to population aging (2). Aging was occurring at a more rapid pace in South Korea compared to that in other countries. There was an eight-year difference between the mean life expectancy (81 years) and disability-adjusted life expectancy (73 years) (3, 4). Since the elderly account for an unprecedentedly high proportion of the Korean population, the Korean society should pay attention to how healthy the elderly were instead of merely focusing on changes of population structure amid elevated life expectancy and rising elderly population.

What factors were affecting an individual’s health? Factors with important effects on health could be broadly classified into demographic, socioeconomic, lifestyle behavior, and social factors (5). These factors might also be classified into internal factors (such as personality and perception about physical deterioration) and external factors (such as loss of social status from retirement, changes in economic level, and changes in regional environment) (6).

Many researchers had particularly focused on individuals’ socioeconomic levels, classifying social class according to occupation, educational level, and income. These studies had confirmed that there were differences in mortality and morbidity across social classes, shedding light on the importance of socioeconomic factors (7, 8).

Working status might also affect health. Compared to regular workers, involuntary non-regular workers had low job and life satisfaction which in turn could have an adverse impact on the overall quality of life, including health (7). Work hour also influenced workers’ health status. Long work hours had detrimental effects on workers’ health. Non-regular workers had longer work hours than regular workers. Because of this, they couldn’t seek timely medical treatment which in turn leads to job stress, ultimately having adverse effects on their health (9).

Population aging was known as a key factor contributing to the elevation of medical expenditure because per capita medical expense in the elderly was higher than that in other age groups. South Korea was experiencing an unprecedentedly rapid aging of the population. Baby boomers born between 1955 and 1963 would enter older adulthood in the coming decades, based on which the aging population was frequently mentioned as a cause of elevated medical expenditure.

However, relevant previous studies had reported that non-demographic causes, as opposed to the aging population, account for a greater proportion of increase in medical expenditure (10). The proportion of demographic caused in the increase of medical expenditure in Korea had been reported to be 10% in an OECD study (11), 13.8% (12), and 28.7% (13).

Despite the fact that population aging was a potential bidirectional factor that increases and suppresses the increase of medical expenditure, few studies had used longitudinal data to confirm that the importance of pre-mortem medical cost changes over time.

Thus, the objective of the present study was to measure aging rate among OECD countries, quantify effects of aging rate on disease patterns in each country, and present and discuss a theoretical model to explain these effects. OECD aging data and disease burden data from the WHO were used to analyze how burden of disease varied according to the level of medical infrastructure (GDP, medical technology, medical support) compared to changes of aging index using a quasi-experimental model known as difference-in-differences (DID) model. By quantifying these effects of aging on disease burden while controlling for differences in healthcare and economic situations in countries with longitudinal data, this study attempted to analyze the pure effect of aging.

## Materials and Methods

This study used OECD Health data and global burden of disease data from the WHO. The OECD annually selected essential statistical indices in various areas including health and asked its 34 member-countries to submit these data. This index, also known as “health data”, included health status, health and medical resources, and health and medical costs. In particular, these data included information that could be used to directly and indirectly estimate the level of medical-related human and material resources. Hence, these data would be useful for controlling for healthcare level or policies of a particular country to measure effects of aging on disease pattern in the corresponding country. Furthermore, the OECD health data provided information about the population aged 65 years or older in 5-year age units, thus enabling the examination of aging rates in each country (14). These country-specific values could be paired with disability-adjusted life year (DALY) values presented by the WHO for analysis (4, 15).

Because the WHO provided DALY values for 2000 and 2012, we aimed to use the amount of changes between these two years. Meanwhile, the purpose of this study was to compare changes in disease burden in relation to changes of the aging index as an international standard. Thus, we computed the aging index for year 2000 and 2012. In addition, burden of disease in early older adults (ages 65–69 years) and in mid and late older adults (ages > 70 yr) were distinguished.

In this study, a difference-in-differences (DID) model was used to analyze effects of aging. A DID model was a quasi-experimental model that enables comparison of pre-test and post-test statuses. Pure effects of aging were identified by confirming difference in aging index between controlled groups with various burden of disease. This study aimed to measure effects of “aging”. Therefore, DID model was used to compare differences in burden of disease in relation to changes in aging index of OECD countries at 2000 and 2012. This difference was deemed as effects of aging on burden of disease.

In this study, the dependent variable was DALY, the sum of years lost due to disease, years of life lost due to death, and years of life lost (YLL) due to death. Time variable as an independent variable was entered as a dummy variable coded as 2000=0 when global disease burden results were first announced and 2012=1, 13 years after the first announcement. Group variable, also an independent variable, was entered as a dummy variable coded as ages 65–69 years=0 and 70 years or older=1. The interaction between time dummy and group dummy was also entered to show the level of aging in consideration of DID.

Based on previous studies on variables with impact on disease burden, the following variables were used as control variables: national gross domestic product (GDP), total medical expenditure of the population, and public health insurance expenditure. These variables were used to control social, medical, and beneficial infrastructures, respectively. With more qualities of infrastructure, the expenditure for medical services and social supports would get larger (Table 1).

**Table 1:** Description of variables

***Variables***		***Description***
Dependent variable	DALY	Lost year due to diseases + Lost year due to early death
	YLL	Lost year due to early death
Independent variable	Dummy of years	2000=0, 2012=1
	Dummy of group	Early old age (year 65∼69)=0
		Mid and late old age (over 70 years old)=1
	Interaction variable	Interaction between year dummy and group dummy
Control variable	GDP	Average of OECD under=0, over=1
	Total health expenditure	Average of OECD under=0, over=1
	Public insurance expenditure	Average of OECD under=0, over=1

## Results

Table 2 showed results of disease burden in relation to levels of aging according to variables for each OECD member country. Compared to those in 2000, DALY and YLL in 2012 were both increased.

**Table 2:** Comparison of OECD members: Burden of disease

			***DALY***				***YLL***			
			***m***	***sd***	***t***		***m***	***sd***	***t***	
Year	2012	Over 70	2917.8	1421.1	248.51^**^	216.25^**^	1600.5	947.8	14.01^**^	192.91^**^
		65–69	2222.1	786.0			1223.6	679.8		
	2000	Over 70	2421.2	982.1	14.04^**^		1029.4	624.6	181.94^**^	
		65–69	1733.9	1102.3			921.7	913.5		
GDP	Under average	Over 70	2317.9	598.9	149.14^*^	243.77	1528.9	513.2	210.71	287.13
		65–69	1907.8	529.1			1324.9	287.3		
	Over average	Over 70	2416.9	791.1	150.66		1611.1	431.3	129.19^*^	
		65–69	2142.0	319.7			1146.5	103.1		
Total health expenditure	Under average	Over 70	2415.8	768.0	143.18	267.44	1875.8	304.2	245.91	318.55
		65–69	2007.4	579.6			1208.3	224.8		
	Over average	Over 70	2241.1	831.1	152.98		1724.6	504.3	256.18	
		65–69	2164,9	1007.8			1422.9	550.1		
Public insurance expenditure	Under average	Over 70	2679.1	1198.2	263.34^**^	256.11^**^	1811.6	336.9	189.5^**^	398.71^**^
		65–69	1977.7	522.4			1011.3	663.6		
	Over average	Over 70	2238.7	364.1	278.19^**^		1219.7	524.2	381.1^**^	
		65–69	1807.8	298.4			998.1	138.1		

ref: * *P*<.01,

**P<.001

The increase of DALY was greater than that of YLL and the increase in the mid- to late older adults group was greater than that in the early older adults group. However, differences of DALY in relation to aging or GDP were not significant. Burden of disease was significantly higher in mid- to late older adults than that in early older adults for countries with below-average GDP whereas YLL was higher in mid- to late older adults than that in early older adults for countries with high GDP.

There were no significant differences in DALY or YLL in relation to aging or total medical expenditure. However, DALY was high among individuals in countries with lower public health insurance expenditure while DALY was the highest in mid- to late older adults in countries with low public health insurance expenditure. Although differences of YLL were not great compared to those of DALY, YLL was the greatest among super-aged (over 70) population in countries with low public health insurance expenditure.

Table 3 showed results of DID regression analysis for differences in aging levels. In an analysis model with DALY and YLL as dependent variables, DALY was positively significant while YLL was negatively significant. This showed that, even after controlling for other variables, the number of deaths from diseases was significantly decreased today compared to that in the past. Meanwhile, the group dummy for aging levels had a significant positive effect on YLL. The interaction term was an important variable that shows DID effects of aging level. It also had a significant positive effect on YLL while it had no effect on DALY. This suggested that YLL significantly increases with increasing aging levels.

With regard to control variables, there were no significant differences in DALY or YLL in relation to GDP or total medical expenditure. However, DALY and YLL were both significantly decreased with increasing public health insurance expenditure.

**Table 3:** Difference in Difference of aging effect on burden of diseases

		***DALY***		***YLL***	
		**b**	**beta**	**b**	**beta**
Constant		431.85	^**^	214.79	^**^
Dependent variable	Year(A)	31.54	.124^**^	86.66	.214^**^
	Group(B)	198.71	.007	168.29	0.231^*^
	AXB	107.22	.076	98.17	0.115^*^
Control variable	GDP	314.52	.064	114.27	0.187
	Total health expenditure	419.14	.021	217.3	0.217
	Public insurance expenditure	−98.47	−0.145^**^	−107.38	−0.004^*^
Model fit	F/ χ^2^	26.947^**^		38.947^**^	
	R square	0.317		0.638	

ref: * *P*<.01,

**P<.001

## Discussion

This study examined the effect of aging levels on burden of disease in OECD member countries using OECD health data (14) and analyzed changes of burden of diseases in the period between 2000 and 2012.

Disease burden increased over time, with more marked increase in the mid- to late older adults than that in the early old (65–69 years old) adults. The increase of YLL due to premature death was greater in the mid- to late older adults than that in the early older adults. In other words, the elderly’s burden of disease increased over time and such increase of disease burden was greater in the population that was elder. Using an econometric model after controlling for relevant variables, results showed that YLL from disease and YLL due to premature death were significantly increased. Meanwhile, the group dummy for aging levels had a significantly positive effect on YLL. The interaction term, an important variable showing DID effects of aging level, also had a significant positive effect on YLL, although it had no effect on DALY. This suggested that YLL significantly increased with increasing aging levels.

To control for the level of a nation’s medical technology and people’s intention to seek treatment for their diseases, we used GDP and medical expenditure as control variables (16, 17). We also used public health insurance expenditure as a control variable because accessibility to medical facility could also influence treatment intention (18). Results of analysis showed that YLL due to premature death increased with increasing levels of aging. This suggested that the increase of YLL drived the increase of disease burden.

Once developing a disease, it was easy for mid- to late older adults to develop other accompanying diseases as well due to their slow recovery rate and immunocompromised state, which increased their burden of disease from death compared to early older adults (16, 17, 19). Among super-olded group, the burden of disease, especially YLL was about three times greater than the effect on total disease of burden. After examining the moderating effect of aging based on the interaction term, results also showed that aging had a significant effect on disease of burden, particularly YLL due to death.

If the increase of YLL due to death were to be relatively greater among more aged elderly compared to less aged elderly as shown in this study, changes of the population structure, burden of disease per age group, and the interaction between the two factors must all be considered when determining the effects of population aging on future medical resources or medical expenditure (20, 21). Further, based on the measurements of the accurate effects of aging, policies should be devised to contribute to stabilizing burden of disease among the elderly population.

## Conclusion

Disease burden increased over time, with more marked increase in the mid- to late older adults population. The increase of YLL due to premature death was greater in the mid- to late older adults population than in the early older adults population. In other words, elderly’s burden of disease increased over time, and the increase of burden of disease was greater in more elderly population. When examined with an econometric model that controls for the relevant variables, it showed that YLL due to premature death were significantly increased.

## Ethical considerations

Ethical issues (Including plagiarism, informed consent, misconduct, data fabrication and/or falsification, double publication and/or submission, redundancy, etc.) have been completely observed by the authors.
